# Reproducible inference of transcription factor footprints in ATAC-seq and DNase-seq datasets using protocol-specific bias modeling

**DOI:** 10.1186/s13059-019-1654-y

**Published:** 2019-02-21

**Authors:** Aslıhan Karabacak Calviello, Antje Hirsekorn, Ricardo Wurmus, Dilmurat Yusuf, Uwe Ohler

**Affiliations:** 1 Max Delbrück Center for Molecular Medicine, Berlin Institute for Medical Systems Biology, Berlin, Germany; 2 Department of Biology, Humboldt University, Berlin, Germany; 3 Department of Computer Science, Humboldt University, Berlin, Germany

**Keywords:** ATAC-seq, DNase-seq, Footprinting, Bias correction, Reproducibility

## Abstract

**Background:**

DNase-seq and ATAC-seq are broadly used methods to assay open chromatin regions genome-wide. The single nucleotide resolution of DNase-seq has been further exploited to infer transcription factor binding sites (TFBSs) in regulatory regions through footprinting. Recent studies have demonstrated the sequence bias of DNase I and its adverse effects on footprinting efficiency. However, footprinting and the impact of sequence bias have not been extensively studied for ATAC-seq.

**Results:**

Here, we undertake a systematic comparison of the two methods and show that a modification to the ATAC-seq protocol increases its yield and its agreement with DNase-seq data from the same cell line. We demonstrate that the two methods have distinct sequence biases and correct for these protocol-specific biases when performing footprinting. Despite the differences in footprint shapes, the locations of the inferred footprints in ATAC-seq and DNase-seq are largely concordant. However, the protocol-specific sequence biases in conjunction with the sequence content of TFBSs impact the discrimination of footprint from the background, which leads to one method outperforming the other for some TFs. Finally, we address the depth required for reproducible identification of open chromatin regions and TF footprints.

**Conclusions:**

We demonstrate that the impact of bias correction on footprinting performance is greater for DNase-seq than for ATAC-seq and that DNase-seq footprinting leads to better performance. It is possible to infer concordant footprints by using replicates, highlighting the importance of reproducibility assessment. The results presented here provide an overview of the advantages and limitations of footprinting analyses using ATAC-seq and DNase-seq.

**Electronic supplementary material:**

The online version of this article (10.1186/s13059-019-1654-y) contains supplementary material, which is available to authorized users.

## Background

The discovery and characterization of *cis*-regulatory elements (CREs) such as promoters, enhancers, and insulators are instrumental in delineating the mechanisms of transcriptional gene regulation. These tissue- and developmental stage-specific regulatory elements reside in nucleosome-free, accessible regions of the genome that are hypersensitive to nuclease attack [1]. Digestion with the nuclease DNase I, coupled to high-throughput sequencing (DNase-seq), was the first established genomic technique to probe such open chromatin regions [2, 3] and was widely applied in research consortia such as ENCODE [4, 5] or the Roadmap Epigenomics [6]. A more recent technique, the assay for transposase-accessible chromatin using sequencing (ATAC-seq), employs Tn5 transposase enzymes that preferentially fragment and tag open regions [7]. Both protocols determine genome-wide chromatin accessibility and can locate distal and proximal CREs.

Transcription factors (TFs) bound at CREs are major regulators of gene expression [8]. As protein-bound DNA is more resistant to cleavage with DNase I, leaving behind protected stretches of nucleotides or shortly “footprints” [9], DNase-seq potentiates the inference of TF-bound locations genome-wide (TF footprinting) [10, 11]. A multitude of TF footprinting methods has been developed to date [12], which can be grouped under three general categories: site-centric, segmentation-based, and integrative site-centric methods. Site-centric methods model footprints specifically for candidate TF binding sites (TFBSs), using the shape or magnitude of the DNase-seq signal around them [13–16]. Segmentation-based methods, on the other hand, scan the DNase-seq signal for footprint-like signatures (e.g., peak-trough-peak pattern) and subsequently match the identified footprints to putative TFs [17–21]. Integrative site-centric methods model bound sites using combinations of diverse features, such as motif match score, sequence conservation, and variable length bins of DNase-seq signal around candidate TFBSs [22–27].

The efforts to assay bound sites genome-wide via TF footprinting have come under scrutiny by studies demonstrating that DNase I cleaves the underlying DNA in a non-uniform manner, where sequence composition dictates the cleavage propensities (also known as sequence bias) [28, 29]. This necessitates the discrimination of actual footprints from footprint-like signal profiles originating solely due to sequence bias [16]. To account for this, a number of TF footprinting tools explicitly model and incorporate the bias background in their models or processing pipelines, by calculating the ratio of observed to expected DNase cuts for short sequences of fixed length [12, 15, 20]. 6-mers have been the primary choice, as they capture enough variation to represent the bias [16], in line with the finding that the main sequence information content around a DNase cut site is confined to the flanking three nucleotides on either side [28]. Open chromatin regions [12, 16, 20] or DNase-seq experiments conducted on deproteinized genomic DNA [12, 15] have been used to infer these 6-mer cleavage propensities.

Recent efforts have explored the feasibility of TF footprinting with ATAC-seq [24, 25, 30]; however, this is not yet studied as extensively as for DNase-seq. Furthermore, like DNase I, Tn5 transposase is reported to have specific sequence preferences [30, 31], but the effect of this on ATAC-seq TF footprinting efficiency is not systematically investigated. It is thus unclear whether the same set of sites would be identified as footprints using ATAC-seq and DNase-seq in a comparative setting. Here, we infer footprints using data obtained from DNase-seq and a modified ATAC-seq protocol in the same cell line, taking the enzyme-specific sequence biases into account, and we show that despite the difference in footprint shapes, the locations identified as bound are in concordance. We report that TF footprinting efficiency is closely linked to clear discrimination of the footprint from the background, which is dependent on the enzyme-specific biases and the sequence content of the TFBSs, making one method more preferable than the other for some TFs, with DNase-seq outperforming ATAC-seq in most cases. Furthermore, we demonstrate that bias correction has a greater impact on footprint model performance for DNase-seq, compared to ATAC-seq. We also address the largely open question on library depth that is required for identification of open chromatin regions and footprints, based on the irreproducible discovery rate (IDR) in conjunction with libraries sequenced to different depths. Our analysis demonstrates that careful consideration of the inherent sequence bias, especially for DNase-seq, and assessment of reproducibility render TF footprinting feasible, even at moderate sequencing depths.

## Results

### A modified ATAC-seq protocol decreases mtDNA contamination and improves agreement with DNase-seq

**Fig. 1:**
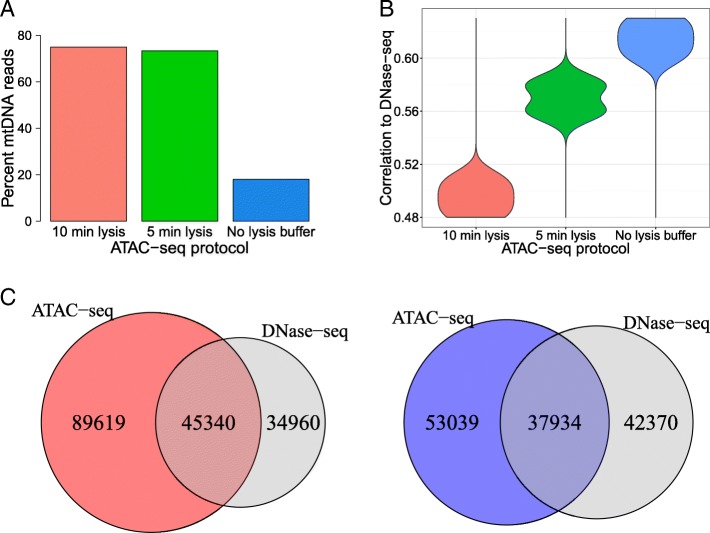
Generating ATAC-seq libraries without the usage of lysis buffer increases agreement with DNase-seq. **a** Percentage of all reads that align to the mitochondrial genome in K562 ATAC-seq libraries generated with the published protocol (10 min lysis), shorter lysis (5 min lysis), or without using lysis buffer (no lysis buffer). **b** Agreement of these libraries with all K562 DNase-seq libraries as measured by Pearson correlations of read counts in 100 bp bins genome-wide. **c** Overlap of peaks found in K562 DNase-seq data with peaks in ATAC-seq data generated using the published protocol (left) and peaks in ATAC-seq data generated without using lysis buffer (right)

Early ATAC-seq libraries generated with the original protocol have large numbers of reads mapping to mitochondrial DNA (mtDNA) that need to be discarded, which severely impact the final library depth [7]. For an ATAC-seq library where we followed this protocol, we made the same observation in K562 cells, with 75% of the reads mapping to mtDNA (Fig. 1a, Additional file 1: Table S1). To decrease the mtDNA contamination, we evaluated two different approaches: decreasing the time of cell lysis to 5 min in lysis buffer (from the original 10 min) and eliminating the lysis buffer step altogether by proceeding directly to the transposition reaction. Of these, particularly, the approach where no lysis buffer was used led to a substantial improvement, with only 18% of the reads mapping to mtDNA in this library (Fig. 1a, Additional file 1: Table S1), in line with previous reports [32]. Avoiding the detergent lysis may help the mitochondrial membranes to stay intact, with other forces such as osmotic pressure being adequate to permeabilize the nuclear membrane.

To adequately quantify the protocol-related differences of ATAC-seq vs. DNase-seq, we also generated a single-hit DNase-seq library in K562 cells and compared this alongside three other publicly available single-hit DNase-seq datasets (Additional file 1: Table S2) with the ATAC-seq libraries. Avoiding the usage of lysis buffer also increased the read-level agreement between the two experimental approaches (Fig. 1b, Pearson correlations of read counts in 100 bp bins; Additional file 1: Figure S1A). This effect was already partially visible in data from the short lysis protocol. To investigate whether this observation is also reflected at the region level of open chromatin, we called peaks with JAMM [33] and identified the set of concordant peaks using the irreproducible discovery rate (IDR) pipeline for DNase-seq data where replicates were available (see the “Methods” section) [34]. Using the peak signal values for ranking, at the stringent 0.01 IDR threshold, we found 80,300 JAMM-IDR peaks for DNase-seq. We also called peaks with JAMM in the ATAC-seq datasets; since replicates were not available for these libraries, the IDR procedure was not applied here. We found 134,761 and 90,973 peaks for the original protocol and the modified protocol with no lysis buffer usage, respectively. Compared to the original protocol, the open regions identified with the modified protocol are more TSS-proximal, with higher GC content, and, in line with previous reports [32], have a modestly reduced signal-to-noise ratio (Additional file 1: Figure S1B). The ATAC-seq peaks found with the original and modified protocols had 45,340 (Fig. 1c, left) and 37,934 (Fig. 1c, right) overlaps to DNase-seq peaks, respectively. Using an extended unfiltered set of open regions as background for Fisher’s exact test, both overlaps were found to be highly significant (*p* value < 2.2e−16), with a slightly higher odds ratio for the modified protocol (13.15 vs. 10.75). This improved agreement at the open chromatin region level, albeit moderate, provided further support that avoiding detergent lysis increases the concordance between ATAC-seq and DNase-seq.

### Open chromatin regions are found reliably at moderate library depths

**Fig. 2:**
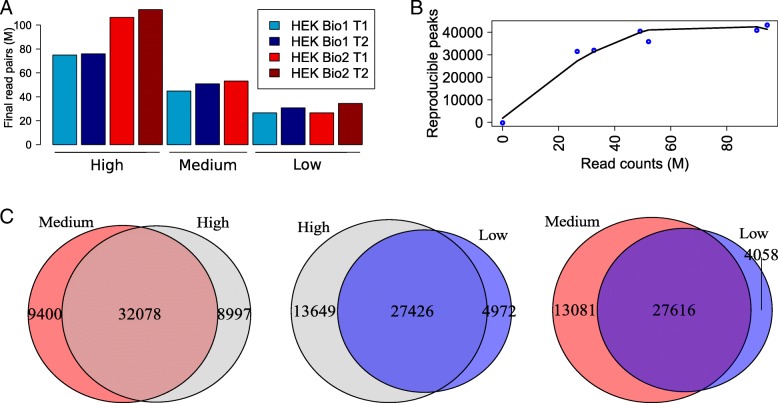
The task of finding open chromatin regions saturates at medium depth. **a** Number of reads after processing in the 11 HEK293 ATAC-seq libraries with different library depths. The 2 biological replicates are shown in blue and red, with the shades representing the technical replicates. **b** Numbers of reproducible peaks found with the JAMM-IDR strategy at different depths. **c** The overlaps between 1 set of peaks in **b** shown for high vs. medium (left), high vs. low (middle), and medium vs. low sets (right)

The library depth of next-generation sequencing protocols that is required for a given downstream application is not always clear, especially when the regions of interest are not as clearly defined as, for example, protein-coding genes. To investigate the effect of library depth on uncovering open chromatin regions, we generated 11 ATAC-seq libraries with different depths in HEK293 cells using the protocol with no lysis buffer (4 high-, 3 medium-, and 4 low-depth libraries, Fig. 2a and Additional file 1: Table S1). The individual libraries were derived from 2 biological replicates. To obtain the highest possible depth representing these 2 samples (> 300,000,000 read pairs each), all technical replicates were merged and denoted by “combined ATAC-seq replicates.” Alongside the ATAC-seq experiments, we generated a single-hit DNase-seq library in HEK293 cells and additionally downloaded and processed two publicly available single-hit DNase-seq replicates (Additional file 1: Table S2). We observed strong positive correlations between all ATAC-seq and DNase-seq libraries at the level of genome-wide read counts (0.62–0.77 Pearson correlations of read counts in 100 bp bins; Additional file 1: Figure S2), and JAMM-IDR peaks called for the combined ATAC-seq and DNase-seq replicates showed again a significant overlap (Additional file 1: Figure S3A).

We then investigated to what extent the individual ATAC-seq libraries sequenced at different depths could capture the open chromatin regions uncovered by the combined replicates. To this end, libraries of similar depth from different biological replicates were matched in a pairwise manner to get JAMM-IDR peaks (Additional file 1: Table S3). This resulted in six total peak sets, corresponding to two of each of high-, medium-, and low-depth library comparisons. Similar numbers of peaks were found at high and medium depth, with a slight decrease at low depth (Fig. 2b, Additional file 1: Figure S3A). Additionally, these peak sets displayed notable agreement among themselves and with the peaks of the combined ATAC-seq dataset (Fig. 2c, Additional file 1: Figure S3A). These observations suggested near saturation for the task of defining open chromatin regions, even though none of the libraries was at saturation at these depths (Additional file 1: Figure S4). Moreover, these six IDR peak sets showed 63% to 72% overlap with the peaks of the DNase-seq data, which exceeded the 61% observed for the combined ATAC-seq data (Additional file 1: Figure S3A); even though a higher number of peaks was found in the combined dataset, IDR analysis of the individual datasets led to more reproducible subsets of the total pool. In support of this, the peaks found in the combined ATAC-seq dataset that did not overlap any of the peaks in the six individual sets were predominantly low-signal, distal regions (Additional file 1: Figure S3B). Taken together, replicate libraries of low to medium depth of 25–50 million reads were sufficient for reliable identification of open chromatin regions in human cell lines.

### Sequence bias of ATAC-seq deviates from that of DNase-seq

**Fig. 3:**
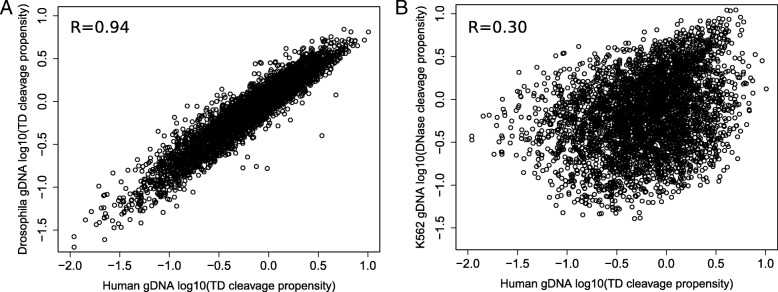
The sequence bias of the Tn5 transposase is distinct from that of DNase I. **a** Comparison of Tn5 transposition propensities of all 6-mers (log10 scale) in two libraries generated using deproteinized genomic DNA from human (YH1) and *D. melanogaster*. **b** 6-mer transposition propensities in the human library compared to cleavage propensities of DNase inferred previously from a single-hit DNase-seq experiment using deproteinized genomic DNA from K562 cells

A multitude of studies has explored the efficacy of transcription factor footprinting with DNase-seq. These studies have demonstrated that the DNase I enzyme cleaves genomic DNA in a non-random fashion, where it has different cut propensities for different sequences, and this sequence bias has adverse effects on the quality of footprinting when left uncorrected [16]. Our lab has previously published a site-centric computational footprinting tool where 6-mer DNase bias has been incorporated into the model to estimate the bias background in a multinomial mixture framework [15]. In order to gain insights into the sequence bias of ATAC-seq data, we calculated the 6-mer cleavage propensities of the Tn5 transposase, using available data from libraries generated by Tn5 transposition on deproteinized genomic DNA [31] (Additional file 1: Table S2). Comparison of the cleavage propensities in libraries generated using human genomic DNA vs. *D*. *melanogaster* genomic DNA revealed very similar results (Fig. 3a, Pearson correlation 0.94), indicating that the Tn5 transposase has specific sequence preferences which are consistent in data from the two species. The dynamic range of this bias is on the same order of magnitude as for DNase bias [15]. We next asked how the sequence preferences of the Tn5 transposase compare to those of DNase I. Using values inferred previously from a single-hit DNase-seq experiment of deproteinized K562 cells [15], we observed this correlation to be fairly low (Fig. 3b, Pearson correlation 0.30). This indicated that these enzymes have largely distinct sequence biases.

### ATAC-seq and DNase-seq generate different footprint shapes for the same factor

**Fig. 4:**
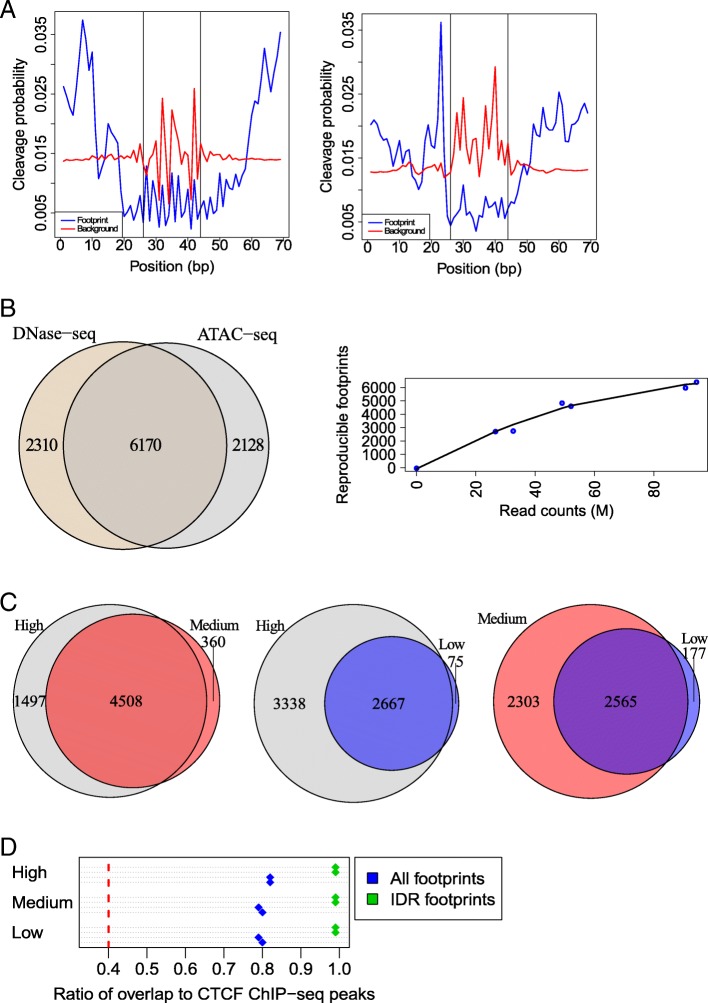
The number of reproducible footprints scales with library depth. **a** CTCF footprints inferred from HEK293 ATAC-seq data (left) and DNase-seq data (right). Vertical lines depict the edges of the motif match. **b** Overlap between reproducible CTCF footprints in the HEK293 DNase-seq and combined ATAC-seq replicates, found using the FLR-IDR strategy. **c** Numbers of reproducible CTCF footprints in HEK293 ATAC-seq datasets at different depths. **d** The overlaps between one set of footprints in **c** shown for high vs. medium (left), high vs. low (middle), and medium vs. low sets (right). **e** The ratio of reproducible CTCF footprints (IDR footprints) or all CTCF motif regions with positive footprint scores (all footprints) that overlap CTCF ChIP-seq peaks, in all six individual sets at different depths (Additional file 1: Table S3). Red dashed line indicates this ratio for all considered CTCF motif sites

In order to systematically examine how ATAC-seq compares to the more established DNase-seq method in transcription factor footprinting, we first focused on CCCTC binding factor (CTCF), a factor with a well-known, high information content binding site with substantial available ChIP-seq data including in HEK293 cells (Additional file 1: Table S4). We scanned the human genome for matches to the CTCF binding model obtained from the JASPAR database (Additional file 1: Table S5). As aggregate signal across all candidate CTCF motif matches is expected to be a mixture of footprint (bound sites) and background (unbound sites), our method [15] was applied to infer the bound subset by modeling the shapes of the CTCF footprints in the DNase-seq and combined ATAC-seq replicates. The shape of the aggregate signal at sites that overlap CTCF ChIP-seq peaks was used to initialize the footprint model. The background was modeled using protocol-specific bias values. The resulting footprint and background profiles revealed marked differences between ATAC-seq and DNase-seq (Fig. 4a, left and right, respectively). Most notable was a wider region of protection in the ATAC-seq data, in line with a previous study [31] which reported that the Tn5 transposase dimer needs circa 30 nucleotides to bind DNA and that cleavage occurs in the central 9 nucleotides. Another difference concerned the background profiles, attributable to the distinct sequence preferences of these two enzymes. In short, from the same set of CTCF motif matches, different footprint and background models were learned using ATAC-seq and DNase-seq datasets.

### Footprinting using ATAC-seq and DNase-seq uncovers common bound sites

This observation led to the question whether the same sites would be identified as bound by a transcription factor when using ATAC-seq and DNase-seq in the same cell type. Using the protocol-specific footprint and background models learned for CTCF, we calculated the footprint scores for all considered motif matches, as the log odds of footprint vs. background per site (footprint log-likelihood ratio (FLR), see the “Methods” section). The FLR is thus derived in a protocol-specific manner, solely from the single-nucleotide resolution signal around motif sites, without relying on additional features, and it accounts for sequence bias, making it an ideal metric to compare the footprints from the two protocols. As a positive FLR indicates a higher probability of being bound vs. unbound, we selected the motif matches that had a positive FLR in both replicates of the assayed method. We again used IDR to find the reproducible subset of CTCF footprints among these sites, ranked by FLR (FLR-IDR, see the “Methods” section). Following this methodology for the combined ATAC-seq replicates, 12,651 motif sites had positive FLRs in both replicates, of which 8298 were found to be reproducible by FLR-IDR (Additional file 1: Figure S5A). For the DNase-seq replicates, of the 13,592 sites with positive FLRs, 8480 were reproducible. Nearly all of the reproducible footprints of ATAC-seq and DNase-seq overlapped CTCF ChIP-seq peaks (98% and 96%, respectively; Additional file 1: Figure S5A). Furthermore, these reproducible footprints from the two experimental protocols were also concordant, with 6170 sites (74%) overlapping (Fig. 4b). This analysis of ATAC-seq and DNase-seq data thus identified many common sites as bound, despite the difference in footprint shapes.

We next investigated the individual contributions of bias modeling and replicates to this increased concordance and accuracy. The contribution of the replicates comes from the application of IDR as mentioned above, which creates a systematic way to find relevant cutoffs for the footprint score. To elucidate the contribution of bias, we first trained CTCF footprint models in the combined ATAC-seq and DNase-seq replicates, as outlined above, but using a uniform background, which is equivalent to no bias correction (see The “Methods” section). We then compared the sensitivity-specificity trade-off between the bias-corrected and uncorrected models, for both DNase-seq and ATAC-seq (Additional file 1: Figure S5B; IDR thresholds agreed well with observed specificity). Bias correction increased the sensitivity of only DNase-seq, and the specificity was not affected for either method. Moreover, correcting bias in DNase-seq had a greater impact than correcting bias in ATAC-seq on the CTCF footprint score correlations between the two experimental methods (Additional file 1: Figure S5C). To investigate this further, we trained footprint models with and without bias correction for three additional transcription factors (MAZ, REST, and YY1) with available ChIP-seq data in HEK293 cells. We compared the model performances using the area under the precision-recall curve for both ATAC-seq and DNase-seq (Additional file 1: Figure S5D). This again revealed a larger impact of bias correction on model performance for DNase-seq compared to ATAC-seq, including a rare case in which correction leads to decreased performance. This observation may result from the factor not leaving a footprint due to a short residence time on chromatin and thus true bound sites showing signals that resemble the bias background. In any case, DNase-seq bias correction had a more pronounced effect on TF footprinting than ATAC-seq bias correction.

### Number of reproducible footprints scales with library depth

Previous studies that inferred cell type-specific TF binding site annotations from DNase footprint data typically used very large datasets (with hundreds of millions of reads per cell type) [10, 17]. To investigate the feasibility of footprinting at lower library depths, we next conducted the analysis on the 11 individual ATAC-seq libraries. We used the same setup for pairwise comparisons as for peak calling (Additional file 1: Table S3), this time to find reproducible CTCF footprints at different library depths. Even though the numbers of motif matches that had positive footprint scores were in the same range for all analyzed pairs, the numbers of reproducible footprints gradually declined with decreasing depth (Fig. 4c, Additional file 1: Figure S5A). This indicated that, unlike peak calling, footprinting efficiency did not saturate and rather followed the library complexities at these depths (Additional file 1: Figure S4). However, the footprints at distinct depths had substantial overlaps with each other and also constituted almost perfect subsets of the footprints found in the combined ATAC-seq data (Fig. 4d, Additional file 1: Figure S5A). Moreover, these reproducible footprint sets consistently showed 99% overlap with CTCF ChIP-seq peaks, compared to around 80% when considering all motif sites with positive FLRs (Fig. 4e). Taken together, even though deeper sequencing is beneficial to footprinting coverage, the assessment of reproducibility enables finding smaller but equally reliable sets of footprints at lower depths.

### Properties of footprinting apply to larger sets of transcription factors

To elucidate whether the previous observations would also apply more generally beyond CTCF, we conducted the footprinting analysis on other factors. The limited availability of ChIP-seq data in HEK293 cells motivated an experimental setup to learn the footprint shapes in K562 cells, where ChIP-seq data is more abundant (Additional file 1: Table S4), and use these models to find footprints in HEK293 cells. To this end, all ATAC-seq data in K562 cells were merged to get adequate depth (Additional file 1: Table S1), and among the K562 DNase-seq datasets, the second ENCODE replicate was chosen (Additional file 1: Table S2). As proof of principle, we first confirmed that the CTCF footprint shapes were almost identical to those learned from HEK293 data (Additional file 1: Figure S6A). We then learned footprint models from K562 data for 19 additional transcription factors with available ChIP-seq data (Additional file 1: Tables S4 and S5). For a subset of these factors, namely NRF1, CREB1, and USF1, the footprint shapes reflected the expected protection pattern in both ATAC-seq and DNase-seq data; in line with the previous observations from CTCF motif regions, the ATAC-seq footprints displayed a wider region of protection compared to the DNase-seq footprints (shown for NRF1 in Additional file 1: Figure S7A). The footprint scores (FLR) for these three factors and CTCF were in close correspondence with the associated ChIP-seq signal values in K562 cells, conferring further confidence in these footprint models (Additional file 1: Figure S6B-E). Thus, we used these models to identify bound sites reproducibly with the FLR-IDR strategy in HEK293 cells. As for CTCF, reproducible footprints were found to be concordant between DNase-seq and combined ATAC-seq replicates; at the level of individual HEK293 ATAC-seq datasets, library depth and the numbers of reproducible footprints showed again a strong dependency (shown for NRF1 in Additional file 1: Figure S7B and C, respectively). As the observations could be replicated for multiple factors, these results likely provide insights into the general properties of the footprints.

### Protocol-specific sequence biases influence footprinting efficiency

**Fig. 5:**
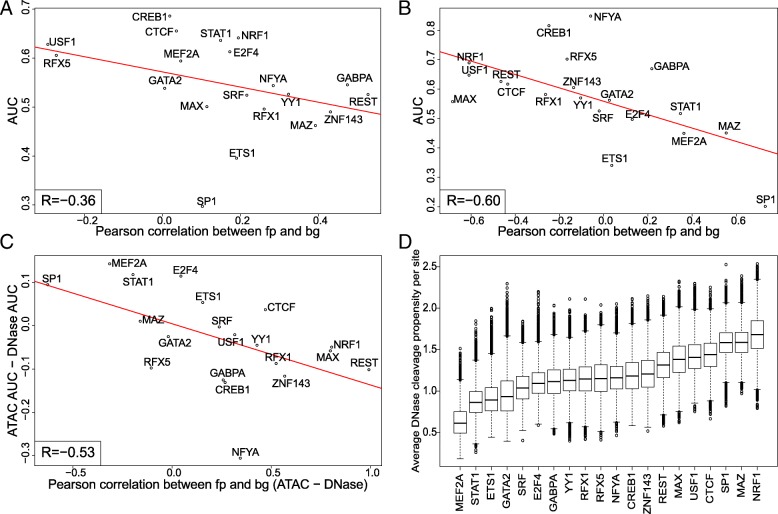
TF footprinting accuracy is linked to clear discrimination of footprint from the background. **a**, **b** AUCs vs. footprint-background model similarities in (**a**) ATAC-seq data and (**b**) DNase-seq data. **c** Difference in AUCs (ATAC-DNase) vs. difference in footprint-background model similarities (ATAC-DNase). **d** Average DNase I cleavage propensities over candidate TFBSs for all 20 assayed factors

Strong footprints that were concordant in both ATAC-seq and DNase-seq data were only found for 4 of the 20 assayed factors. For most factors, clear footprints were observed in 1 of the experimental methods, but not the other. Therefore, we asked whether the distinct sequence biases of the 2 methods play a role in the factor-dependent performance of footprinting. To get a continuous measure for performance (as opposed to the discrete visual assessment of footprint shapes), for all TFs in both experimental settings, we calculated the area under the receiver operating characteristic curve (AUC), ranking candidate sites by FLR and considering those that overlap ChIP-seq peaks to be true binding sites. In order to assess how the performance is linked to the relationship between the footprint and background models, the Pearson correlations between these 2 models (e.g., footprint-background model similarities) for each TF were calculated and compared to the AUCs. The AUCs negatively correlated with the footprint-background model similarities in both ATAC-seq and DNase-seq datasets (Fig. 5a, b, correlations of − 0.36 and − 0.6, respectively), indicating that when a footprint model is clearly distinguished from the background, it is more likely to explain the transcription factor binding accurately. Moreover, the differences per TF between ATAC-seq and DNase-seq datasets for these 2 measures (AUCs and footprint-background model similarities) also had a negative correlation (− 0.53, Fig. 5c), suggesting that the experimental protocol which achieves better separation between the footprint and background components is also performing better for a given TF. Overall, DNase-seq footprinting had a clear advantage over ATAC-seq-derived footprints (cf. Additional file 1: Figure S8A, which compares the area under the precision-recall curve values).

As the background component is derived directly from sequence bias and given our previous observation that DNase-seq bias correction shows a stronger positive effect compared to ATAC-seq bias correction, we once again explored the role of bias more explicitly. In particular, two of three factors for which ATAC-seq outperformed DNase-seq, MEF2A, and STAT1 had the lowest DNase I cleavage propensities (e.g., sequence bias) over their motif regions, among all assayed factors (Fig. 5d), whereas the Tn5 transposition propensities for these factors were average (Additional file 1: Figure S8B). Therefore, the background models learned from DNase bias for these factors had footprint-like shapes, impeding the clear separation between the two components, and thus explaining the poor performance of DNase-seq (shown for MEF2A in Additional file 1: Figure S8C). The equivalent scenario was not as clear to observe for ATAC-seq, possibly due to the difference in the efficiency of bias modeling (see the “Discussion” section). In summary, due to the distinct sequence biases of ATAC-seq and DNase-seq, the sequence content of transcription factor binding sites can influence footprinting efficiency in a protocol-specific manner.

## Discussion

DNase-seq has been widely used to assay open chromatin regions and TF footprints. The emergence and increasing use of ATAC-seq necessitate a systematic comparison of the two methods, especially for TF footprinting. Here, in a comparative setting, we have shown that although the two methods have distinct sequence biases and generate different footprint shapes for the same TF, the sites they identify as bound are largely in agreement. However, the sequence content of TFBSs combined with protocol-specific sequence biases impacts footprinting efficiency for some TFs, leading to larger differences for these factors and making one method preferable to the other.

There are opposing views on the library depth required for TF footprinting. Whereas some studies require at least 200 million reads [10], others demonstrate efficient TF footprinting at moderate sequencing depths (50–60 million reads) [23, 30], in agreement with our results. These moderate numbers were reported for both segmentation-based [30] and integrative site-centric [23] tools, challenging the view that these approaches have different depth requirements [10]. To get the highest possible depth, pooling all replicates has been a common practice in TF footprinting studies. However, our results indicate that keeping the replicates separate to assess reproducibility may lead to more accurate footprint predictions. This is especially relevant for low-depth libraries, where this approach enables finding reliable subsets of the total footprint pool.

Although the sequence bias of DNase I is well characterized, there is still no consensus about the benefits of bias correction for TF footprinting. Whereas some studies report increased accuracy upon bias correction [12], others do not make this observation [23]. One explanation for this might be the different approaches to DNase signal processing and TF footprinting. Methods that extensively smooth the signal or use features that diverge from single-nucleotide resolution (e.g., binned signal) might be less affected by bias. Since our method has a single-nucleotide resolution, we have used protocol-specific biases to model the background in our TF footprinting approach, and we could demonstrate significant improvements on footprinting when using bias correction on DNase-seq data. While ATAC-seq footprinting showed also promising results on par with DNase-seq in HEK293 data (Additional file 1: Figure S5D), its performance in K562 data was significantly lower for almost all factors (Additional file 1: Figure S8A). Here, ATAC-seq outperformed DNase-seq only for three factors, two of which had low average DNase I cleavage propensities over their motif regions that resulted in a footprint-like background profile. The opposite was not as clear, i.e., for factors where DNase-seq outperformed ATAC-seq, the average Tn5 cleavage propensities over the motif regions were not consistently at the lower end of the spectrum. Furthermore, the range of average cleavage propensities over all TFs was narrower for Tn5 (Fig. 5d vs. Additional file 1: Figure S8B).

Recent studies have proposed several Tn5 bias correction methods, and in order to rule out that this observation resulted from our 6-mer-based approach (see the “Methods” section for a detailed explanation of our approach), we used a different bias correction, in which a 17-bp-long gapped *k*-mer with eight meaningful positions is used to correct ATAC-seq data [35]. This more sophisticated bias correction method did not improve the footprint model performance (Additional file 1: Figure S8D). Taken together, correcting for Tn5 sequence bias does either not have a strong impact on ATAC-seq footprinting or neither of the approaches we used is comparable in its impact to DNase-seq bias correction.

## Conclusions

Our comparative analysis clearly confirms previous reports that DNase cleavage bias might render footprints of some factors “invisible” and that, in general, performance to identify footprints can vary significantly across assays and TFs. While an effective footprinting for all TFs may in principle be achieved through a combination of assays with different sequence biases, our results do not suggest ATAC-seq for this purpose, due to its reduced performance; although, it is possible to achieve better performance in deeper datasets as exemplified by our HEK293 data. Finally, in contrast to previous studies that reported no correlation between ChIP-seq signal values and footprint scores [20], we have previously observed and now observe again a strong link between these two measures, implying that the footprint score we have defined here is a quantitative measure of occupancy. In summary, we expect that the insights gained from this work will provide experimental design and computational analysis guidelines for future TF footprinting studies.

## Methods

### DNase-seq and ATAC-seq experimental procedures and data preprocessing

DNase-seq and ATAC-seq assays were performed on human cell lines, K562 and HEK293 cells. K562 and HEK293 cells were cultured in Iscove’s modified Dulbecco’s medium (IMDM) and Dulbecco’s modified Eagle’s medium (DMEM), respectively, both complemented with 10% fetal bovine serum (FBS) and 1% penicillin/streptomycin.

DNase-seq experiments were conducted on 50 million cells as previously described [36], with the minor modification of using 5′ phosphorylated oligo 1b. Samples digested with 12 U, 4 U, and 1.2 U total DNase I were pooled. Libraries constructed from pooled digests were sequenced on the Illumina HiSeq2500 platform using the single-end sequencing mode with 50-bp reads. The analysis was conducted in line with the official ENCODE DNase-seq pipeline. Specifically, the reads were trimmed to the first 20 bases, as only this portion corresponded to the ends of DNase I-digested fragments, due to the MmeI cleavage step in the protocol. Trimmed reads were aligned to the hg19 build of the human genome, using the Burrows-Wheeler Aligner (BWA) [37], tolerating up to 2 mismatches. Sequences aligning to more than 4 locations were discarded. Further processing was performed to filter out unwanted chromosomes and problematic regions such as alpha satellites. In order to remove PCR artifacts, reads that piled up (≥ 10 reads) at a single base were discarded, if they constituted at least 70% of all reads in the surrounding 30-bp window.

ATAC-seq experiments were performed on 50,000 cells for the K562 samples and 100,000 cells for the HEK293 samples, following the published protocol [7] but increasing transposition time from 30 min to 1 h for all samples. In addition, lysis conditions were varied in different experiments. For the K562 sample denoted “10 min lysis,” cell lysis was performed via a 10-min centrifugation in lysis buffer, as described in the original protocol [7]. For the K562 sample denoted “5 min lysis,” a shorter lysis of 5 min was used. For the K562 sample denoted “no lysis buffer” and all HEK293 samples, the centrifugation in lysis buffer step was omitted altogether, and the cell pellets were taken directly to the transposition reaction. Libraries were sequenced on the Illumina HiSeq2000 platform, with 100-bp paired-end reads. Since fragments as short as 38 bp were expected, adapter sequences were trimmed from the 3′ end of the reads. Specifically, matches of any length to the reverse-complemented Nextera Transposase Adapters (CTGTCTCTTATACACATCTGACGCTGCCGACGA, CTGTCTCTTATACACATCTCCGAGCCCACGAGAC) were removed. Trimmed reads were aligned to the hg19 build of the human genome, using bowtie2 [38] with parameter -X set to 1500, to allow correct alignment of paired-end fragments up to 1500 bp. Only the reads that aligned uniquely to a single location were retained, by filtering out the multimappers marked with the XS:i flag in the SAM file. PCR duplicates were removed using Picard (http://broadinstitute.github.io/picard/). Further processing was performed to filter out contigs as well as the Y and mitochondrial chromosomes and retain only the reads that aligned concordantly as a pair within the expected fragment length range (38–1500 bp).

Library complexity and saturation were calculated using the preseq program [39], using the c_curve and lc_extrap functionalities. Correlations of read counts between libraries were calculated using the bamCorrelate bins command of the deepTools suite, with the parameters –corMethod pearson, -bs 100, --fragmentLength 1, and –doNotExtendPairedEnds.

### Peak calling

In order to find open chromatin regions, peak calling was performed on the processed DNase-seq and ATAC-seq datasets using JAMM [33], with parameters -f 1 and -d y. Parameter -f 1 ensured taking only the 5′ ends of the reads into account which corresponded to the actual cleavage/transposition sites. As duplicates were already removed prior to peak calling, parameter -d y was used to keep all processed reads.

Where replicates were available, peaks in agreement between the two replicates were found using the irreproducible discovery rate (IDR) pipeline [34]. Specifically, the “batch-consistency-analysis.r” script of the pipeline was executed using the “signal.value” parameter, ranking the peaks of the two replicates by signal intensity for comparison. The “half.width” and “overlap.ratio” parameters were set to − 1 and 0, respectively, where true peak widths were used without alteration and two peaks were considered to be part of the same region if there was at least 1 bp overlap between them. The number of peaks that were found to be concordant at the stringent 0.01 IDR threshold was noted. Then, JAMM was once again used, this time to call peaks on the two replicates together rather than individually, with the -f 1,1 parameter. In this way, peaks were called where both replicates consistently displayed signal enrichment. This peak set was further truncated using the number obtained from the IDR analysis, resulting in the final JAMM-IDR peaks.

For K562 ATAC-seq datasets, where replicates were not available, reads of the modified dataset with no lysis buffer were randomly subsetted to match the library depth of the original protocol with 10 min lysis, and peaks were called using JAMM as described above, with the addition of the -e auto parameter for automatic estimation of a minimum fold enrichment. These K562 ATAC-seq peak sets were used to infer signal to noise ratios by calculating log2(average signal in the peaks/average signal in the 300-bp upstream and downstream flanking regions).

### Sequence bias of Tn5 transposase

The sequence bias of the Tn5 transposase was calculated in the form of 6-mers, similar to the previous calculations of DNase bias [15]. To this end, libraries generated by Tn5 transposition on deproteinized genomic DNA (see Additional file 1: Table S2) were preprocessed in the same way as ATAC-seq datasets as detailed above. As the 5′ ends of the reads corresponded to the transposition sites, the sequences of all 6-mers centered on these sites were retrieved (e.g., transposition between the third and fourth nucleotides). Occurrences of all these 6-mers in the data were counted, and the relative frequencies were calculated for each. Similarly, background genomic frequencies were calculated by counting all 6-mers present in the mappable portion of the genome. The frequencies observed in the data were normalized to the background frequencies to obtain the final transposition propensities per 6-mer. Deviations from one indicated increased or decreased propensities, thus bias.

The Tn5 dimer cleaves the plus and minus strands with a 9-bp offset, and consequently, most studies analyzing ATAC-seq datasets employ a correction where the reads that align to the plus strand are shifted by + 4, and the reads that align to the minus strand are shifted by − 5 bases, to update the read start sites to represent the center of this 9-bp core sequence. However, we focus on the actual 5′ ends of the reads, as these are the cleavage sites, akin to the DNase I cut sites. Our first reason for this is illustrated in Additional file 1: Figure S9A, adapted from reference [40]. Tn5 shows sequence bias over an extended ~ 20-bp region, centered around the core 9 bp, where the central nucleotide is marked with a star, and the read start is at position 0. The box on the left indicates the 6-mer around the cleavage site for the given read, and the box on the right indicates the 6-mer around the cut site on the opposite strand. Therefore, even though we explicitly correct for the 6-mer around the cut sites for each read, implicitly, this can be thought of as accounting for 12 nucleotides within this extended region with bias. The 6-mer sequences denoted in the 2 boxes are symmetrical, i.e., the sequence in the left box matches the reverse complement of the sequence in the right box, which can be visually assessed in Additional file 1: Figure S9A. This is also confirmed when we derive 6-mer bias values around the cut sites only for plus strand or minus strand reads, from libraries generated by Tn5 transposition on deproteinized genomic DNA (see Additional file 1: Table S2 and Figure S9B). The plus and minus strand-derived bias values show very high correlation, which allows us to use a common set of 6-mer bias values that can be applied to all reads regardless of their strand. As noted above, the conventional + 4/− 5 bp correction brings the cut site to the same position in the plus and minus strand reads, right upstream of the central nucleotide (shown as the line in the middle of the box in Additional file 1: Figure S9C). However, the 6-mers around this corrected cut site (the box in Additional file 1: Figure S9C) are no longer symmetrical for the two strands. In this case, one would either have to model the sequence bias separately for the plus and minus strand reads or perform an unconventional + 4/− 4 correction to make sure that the first base is the central nucleotide for both plus strand and minus strand reads. While these are also reasonable choices, we propose our approach as a simple, viable alternative.

The average Tn5 transposition propensity in a candidate binding site of a given transcription factor was calculated by retrieving and counting all 6-mers associated with the site (without flanks). The counts were multiplied by the Tn5 transposition propensities of the associated 6-mers, summed and normalized by the total number of 6-mers in the site. The same calculation was applied for DNase, using the previously calculated DNase cleavage propensities per 6-mer [15].

### Scanning the genome for candidate binding sites

The SpeakerScan Toolset [41] was used to scan the hg19 build of the human genome with position weight matrices (PWMs), to find candidate transcription factor binding sites (TFBS). PWMs contain expected frequencies for each nucleotide in a per-base fashion, modeling the binding sequence preferences of a given TF. A pseudocount of 0.0005 was added to each frequency in the PWMs, to ensure non-zero entries. At each PWM-sized window in the genome, a TFBS score was calculated, as the log-likelihood of the underlying sequence matching the PWM vs. a background model. The background was modeled with a first-order Markov chain in a 500-bp local window, centered on the considered position. The top scoring 50,000 sites were taken along for transcription factor footprinting in this study. To validate the significance of motif matches for all PWMs, we simulated DNA sequences using the PWM model (positive set) and a background modeled with a first-order Markov chain from hypersensitive sites (negative set) and sampled TFBS scores from these positive and negative sets. For a range of false-positive rates (FPR, up to 1 × 10^−6^), we found the corresponding TFBS scores and reported the one closest to the lowest score in each motif set and the associated FPR as an empirical *p* value. This demonstrated that all our sets included significant motif matches, with the lowest empirical *p* value being 5 × 10^−5^ (Additional file 1: Table S5).

### Identification of transcription factor footprints

Transcription factor footprinting was performed with a site-centric method from our lab as previously described [15]. Specifically, candidate TFBSs were considered with 25 bp flanks upstream and downstream (parameter PadLen = 25). Parameter *k* = 2 was used to model two components, one for the footprint and one for the background. Both components were modeled as multinomials along the considered window size (TFBS + 50 bp), where each value corresponded to the cleavage/transposition probabilities at a given nucleotide. For the footprint component, these probabilities were found by computing the aggregate DNase or ATAC-seq signal (from the 5′ ends of the reads) around the TFBSs that overlap ChIP-seq peaks for that factor and re-estimating the signal via expectation maximization. For the background component, the probabilities were calculated as the signal that would be expected solely due to the protocol-specific bias values, given the sequences around the candidate TFBSs (parameter Background = “Seq”). As we had previously not observed a distinct difference in performance, the background was kept fixed and not re-estimated (parameter Fixed = T). Once both components were learned, footprint scores were calculated for all candidate TFBSs, as the log odds of footprint vs. background (footprint log-likelihood ratio, FLR). To learn footprint models without bias correction, our method was applied as described above, but with a uniform, fixed background model that assumes equal cleavage probabilities at each nucleotide.

The IDR strategy was applied here as well where replicates were available, to find reproducible footprints. To this end, candidate TFBSs with positive FLRs in both replicates were chosen and ranked by FLR. IDR analysis was performed with the same parameters as explained for peak calling, where FLR values replaced signal intensities. Again, the number of sites that passed the stringent 0.01 IDR threshold was noted. Finally, TFBSs were ranked by the average FLR from the two replicates and truncated according to the IDR result. This led to the reproducible FLR-IDR footprints.

Footprint model AUCs (both area under the ROC and precision-recall curves) were calculated by fourfold cross-validation. Briefly, the data was split into four parts, and for TFBSs in each part, FLR was calculated using footprint and background models learned from the other three parts. TFBSs were ranked by FLR, and those intersecting ChIP-seq peaks were labeled as the true positives. The AUCs obtained from the four parts were averaged to obtain the final value. Similarly, sensitivity and specificity measures were also obtained using models trained on three out of four parts of the data and tested on the remaining part.

Correction of Tn5 sequence bias in K562 ATAC-seq data with the seqOutBias [35] software was carried out according to the guidelines provided in the vignette. Specifically, --kmer-mask NXNXXXCXXNNXNNNXXN for plus strand reads and --kmer-mask NXXNNNXNNXXCXXXNXN for minus strand reads were used to correct the signal. The corrected data was then used to learn footprint models with our method, in conjunction with a uniform, fixed background model.

## Additional file

Additional file 1: Figure S1. Read count correlations of all K562 datasets and comparison of K562 ATAC-seq datasets generated with the original (10 min lysis) and modified (no lysis buffer) protocols. Figure S2. Read count correlations of all HEK293 datasets. Figure S3. Analysis of reproducible peaks in HEK293 cells. Figure S4. Library complexity and saturation plots for HEK293 ATAC-seq datasets. Figure S5. Analysis of footprint models learned in HEK293 datasets and assessing the effects of bias correction on footprinting performance. Figure S6. The relevance of the learned footprint models as illustrated by model similarity across datasets and concordance between footprint scores and ChIP-seq signals. Figure S7. Analysis of NRF1 footprints. Figure S8. Method and TF-specific footprinting efficiency. Figure S9. 6-mer bias correction strategy for ATAC-seq datasets. Table S1. General statistics of the ATAC-seq datasets generated in the study. Table S2. Descriptions, accession codes and final read counts for the utilized DNase-seq datasets and libraries generated by Tn5 transposition of deproteinized genomic DNA. Table S3. Scheme for ATAC-seq library comparisons for JAMM-IDR peak calls or FLR-IDR footprint calls. Table S4. ChIP-seq peaks used in the analysis. Table S5. PWM IDs used for genome-wide motif searches. (PDF 3299 kb)
