# Supplementary material for: Reproducible inference of transcription factor footprints in ATAC-seq and DNase-seq datasets using protocol-specific bias modeling
Source: Genome Biol. 2019 Feb 21;20:42. doi: 10.1186/s13059-019-1654-y (PMC6385462; doi:10.1186/s13059-019-1654-y)
Supplement: Supplementary file 1 — Figure S1. Read count correlations of all K562 datasets and comparison of K562 ATAC-seq datasets generated with the original (10 min lysis) and modified (no lysis buffer) protocols. Figure S2. Read count correlations of all HEK293 datasets. Figure S3. Analysis of reproducible peaks in HEK293 cells. Figure S4. Library complexity and saturation plots for HEK293 ATAC-seq datasets. Figure S5. Analysis of footprint models learned in HEK293 datasets and assessing the effects of bias correction on footprinting performance. Figure S6. The relevance of the learned footprint models as illustrated by model similarity across datasets and concordance between footprint scores and ChIP-seq signals. Figure S7. Analysis of NRF1 footprints. Figure S8. Method and TF-specific footprinting efficiency. Figure S9. 6-mer bias correction strategy for ATAC-seq datasets. Table S1. General statistics of the ATAC-seq datasets generated in the study. Table S2. Descriptions, accession codes and final read counts for the utilized DNase-seq datasets and libraries generated by Tn5 transposition of deproteinized genomic DNA. Table S3. Scheme for ATAC-seq library comparisons for JAMM-IDR peak calls or FLR-IDR footprint calls. Table S4. ChIP-seq peaks used in the analysis. Table S5. PWM IDs used for genome-wide motif searches. (PDF 3299 kb) [file 13059_2019_1654_MOESM1_ESM.pdf]

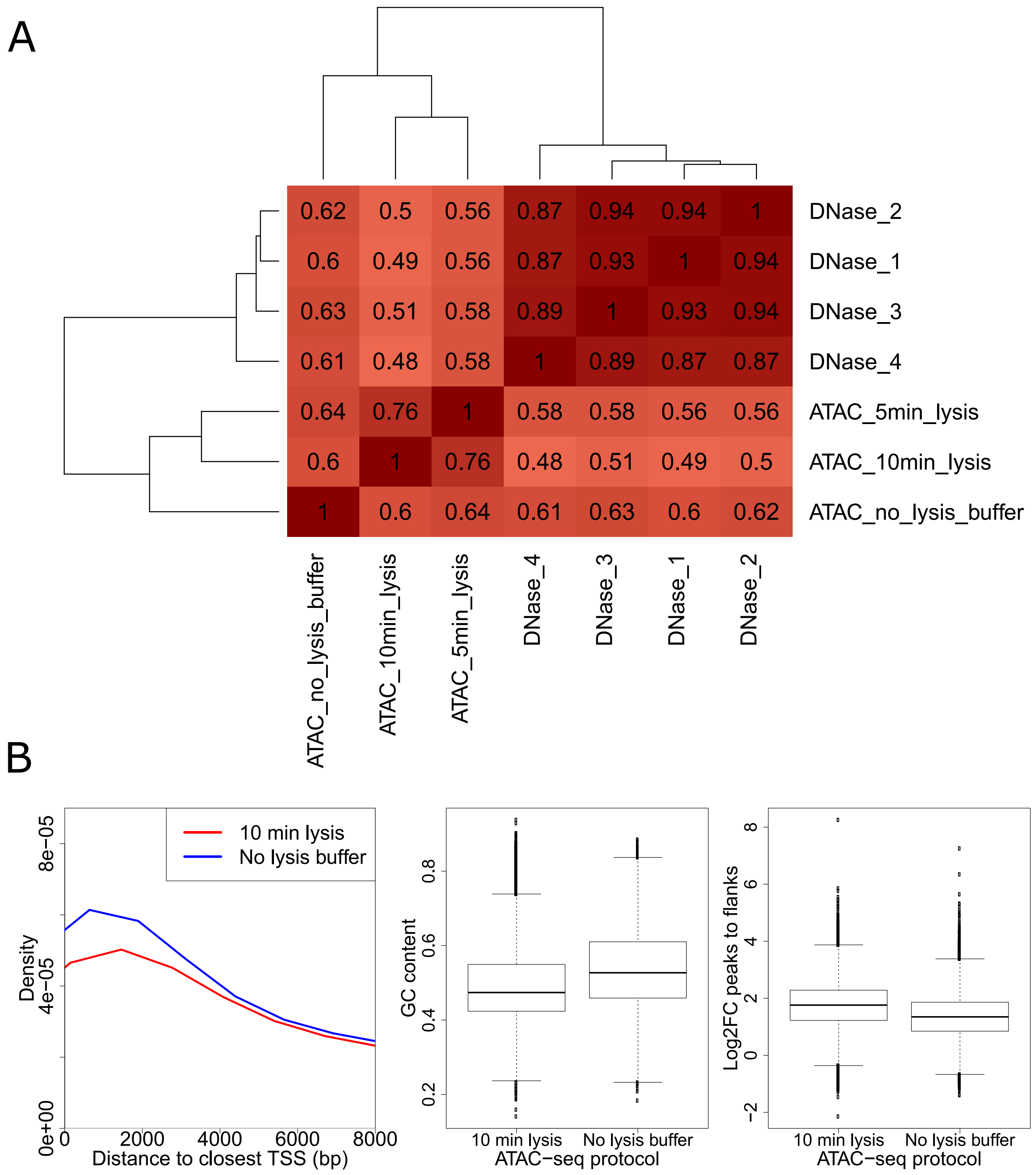

Figure S1: (A) Pairwise Pearson correlations of read counts in 100bp bins genome-wide for all ATAC-seq and DNase-seq datasets in K562 cells. ATAC-seq datasets are labeled with the employed protocol: 10 min lysis (published protocol), 5 min lysis and no lysis buffer. DNase 1-3 are the replicates from the ENCODE project and 4 is the library newly generated for the study, all following the single-hit protocol. (B) Comparison of hypersensitive sites (HSs) found in K562 ATAC-seq datasets generated with the original (10 min lysis) and modified (no lysis buffer) protocols. HSs are compared with respect to distance to the nearest TSS (left), GC content (middle) and log2 fold change of read counts in HSs vs. flanking regions (right).

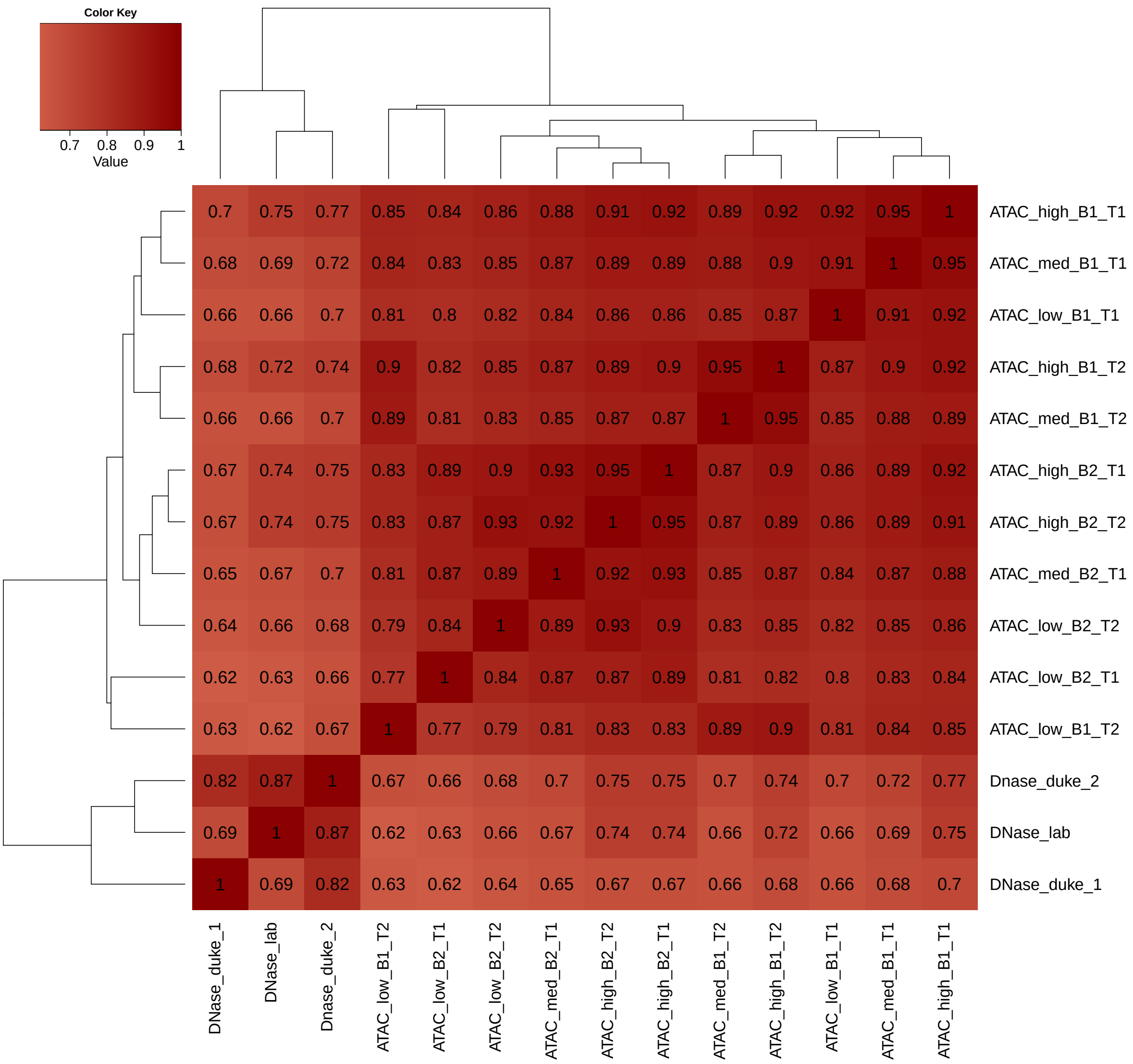

Figure S2: Pairwise Pearson correlations of read counts in 100bp bins genome-wide for the ATAC-seq and DNase-seq datasets in HEK293 cells. All ATAC-seq datasets are generated with the protocol where no lysis buffer is used. The corresponding library depth (high, medium or low), biological (B1 or B2) and technical (T1 or T2) replicate status is indicated. DNase 1 and 2 are the replicates from the ENCODE project and lab refers to the library newly generated for the study, all following the single-hit protocol.

A

	DNase	Combined_ATAC	High_ATAC1	High_ATAC2	Medium_ATAC1	Medium_ATAC2	Low_ATAC1	Low_ATAC2	Number of peaks
DNase		0.61	0.51	0.51	0.52	0.48	0.44	0.44	56019
Combined_ATAC	0.48		0.52	0.53	0.53	0.47	0.43	0.43	68970
High_ATAC1	0.66	0.86		0.77	0.78	0.72	0.67	0.65	41075
High_ATAC2	0.63	0.84	0.74		0.73	0.68	0.63	0.62	43381
Medium_ATAC1	0.66	0.86	0.77	0.76		0.74	0.68	0.66	40647
Medium_ATAC2	0.68	0.87	0.8	0.79	0.84		0.73	0.72	36053
Low_ATAC1	0.72	0.9	0.84	0.83	0.87	0.83		0.77	31674
Low_ATAC2	0.71	0.89	0.82	0.82	0.85	0.81	0.76		32183

B

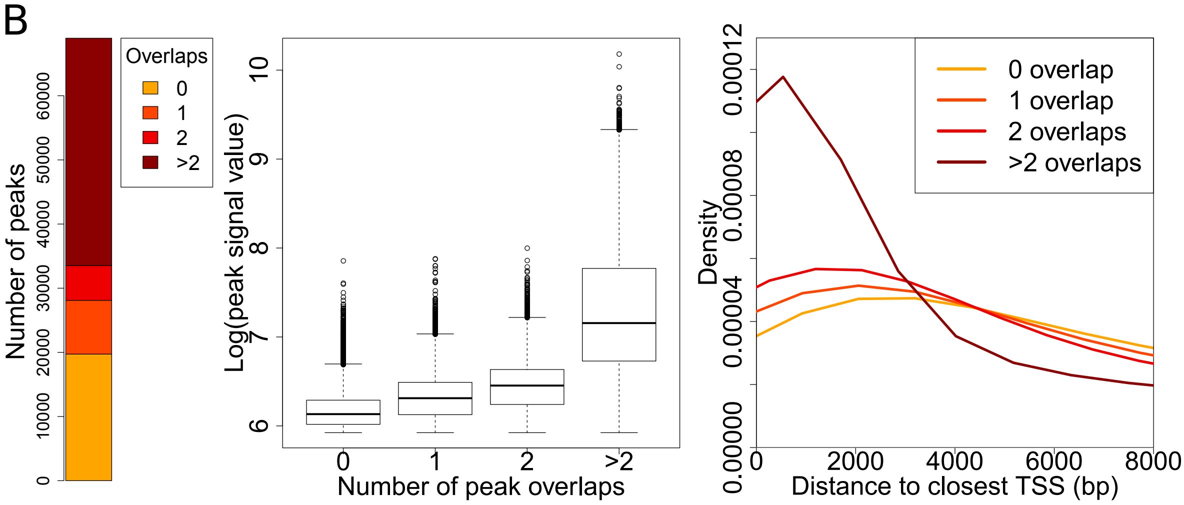

Figure S3: Analysis of reproducible peaks in HEK293 cells. (A) Overlaps between all reproducible JAMM-IDR peaks found in HEK293 DNase-seq and ATAC-seq datasets. The number in each cell represents the ratio of the peaks in the row-dataset that overlap the peaks of the column-dataset. Total numbers of peaks are given on the right. (B) Number of JAMM-IDR peaks in the combined ATAC-seq replicates that overlap the union of peaks from the six individual datasets zero, one, two or more times (left). Peak signal values (middle) and distance to closest TSS (right) are shown for these four groups.

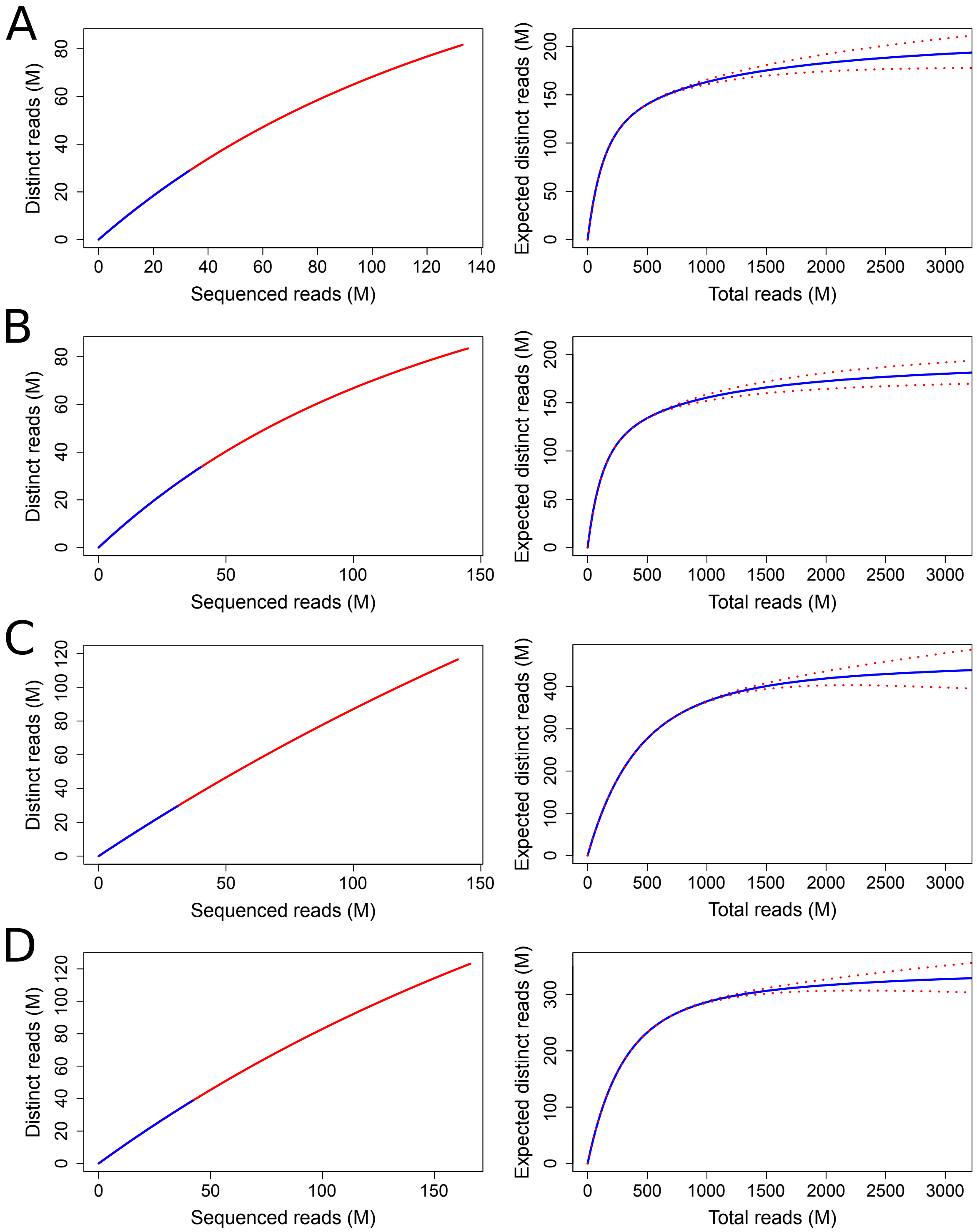

Figure S4: Library complexity and saturation plots for HEK293 ATAC-seq datasets. (A-D) Complexity (left) and saturation plots (right) for (A) biological replicate 1 technical replicate 1 (B1-T1), (B) B1-T2, (C) B2-T1 and (D) B2-T2. Library complexity is shown at high and low library depth levels, in red and blue, respectively.

A

	DNase	Combined_ATAC	High_ATAC1	High_ATAC2	Medium_ATAC1	Medium_ATAC2	Low_ATAC1	Low_ATAC2	Number of reproducible/ total footprints	Overlap with ChIP peaks
DNase		0.73	0.58	0.6	0.48	0.45	0.28	0.28	8480/13592	8151 (96%)
Combined_ATAC	0.74		0.72	0.76	0.58	0.55	0.33	0.33	8298/12651	8114 (98%)
High_ATAC1	0.81	0.99		0.87	0.75	0.7	0.44	0.45	6005/12473	5938 (99%)
High_ATAC2	0.8	0.99	0.82		0.69	0.68	0.41	0.42	6435/12308	6339 (99%)
Medium_ATAC1	0.83	0.99	0.93	0.91		0.82	0.53	0.52	4868/12204	4818 (99%)
Medium_ATAC2	0.83	0.99	0.91	0.94	0.87		0.53	0.55	4634/12107	4585 (99%)
Low_ATAC1	0.86	1	0.97	0.96	0.94	0.9		0.68	2742/11225	2718 (99%)
Low_ATAC2	0.87	1	0.96	0.98	0.91	0.91	0.67		2782/11591	2768 (99%)

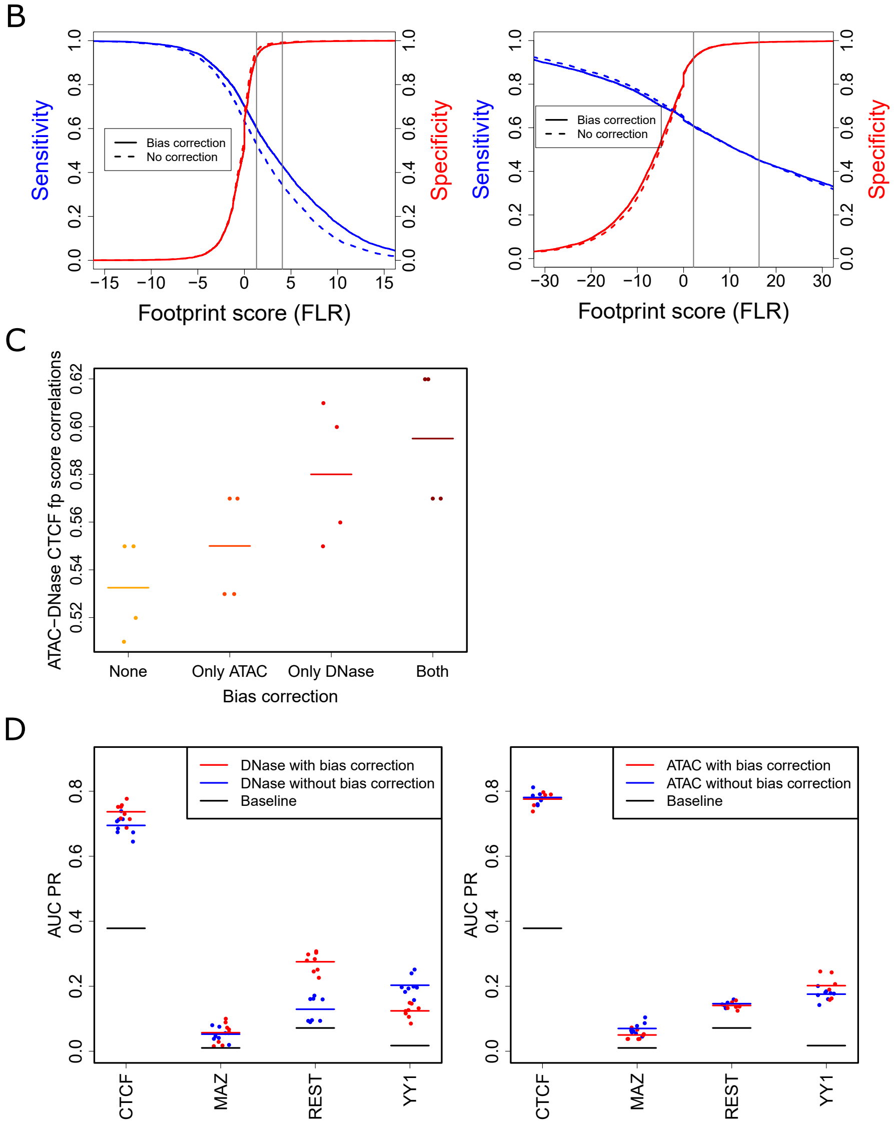

Figure S5: (A) Overlaps between all reproducible FLR-IDR CTCF footprints found in HEK293 DNase-seq and ATAC-seq datasets. The number in each cell represents the ratio of the footprints in the row-dataset that overlap the footprints of the column-dataset. Numbers of footprints and their overlaps with ChIP-seq peaks are given on the right. (B) The relationship between sensitivity and specificity measures of CTCF footprint models found in HEK293 DNase-seq (left) and ATAC-seq (right) datasets with and without bias correction. The vertical lines show the footprint scores that correspond to relaxed and stringent IDR thresholds, 0.1 and 0.01 respectively. (C) Correlations of CTCF footprint scores between HEK293 ATAC-seq and DNase-seq datasets with respect to their bias correction status. (D) Area under the precision-recall curve of footprint models learned for four factors (CTCF, MAZ, REST, YY1) in HEK293 ATAC-seq and DNase-seq datasets.

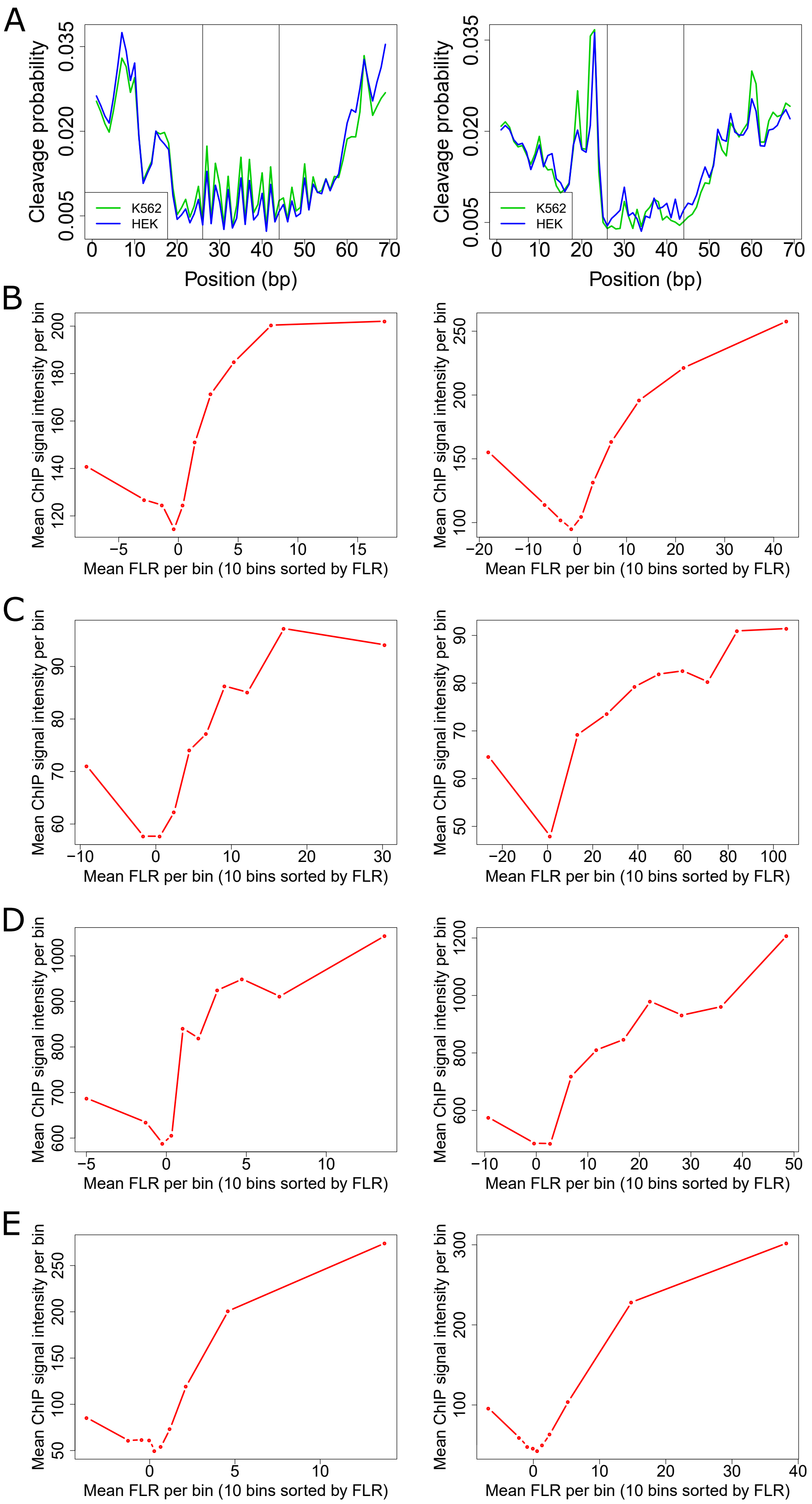

Figure S6: The relevance of the learned footprint models. (A) Highly similar CTCF footprint profiles in HEK293 and K562 ATAC-seq (left) and DNase-seq (right) datasets. (B-E) Concordance between ChIP-seq signal intensities and footprint scores (FLR) in K562 ATAC-seq (left) and DNase-seq (right) data for (B) CTCF, (C) NRF1, (D) CREB1 and (E) USF1. Motif sites that overlap ChIP-seq peaks are divided in ten bins according to FLR. The mean ChIP-seq signal intensity and FLR is plotted for each bin.

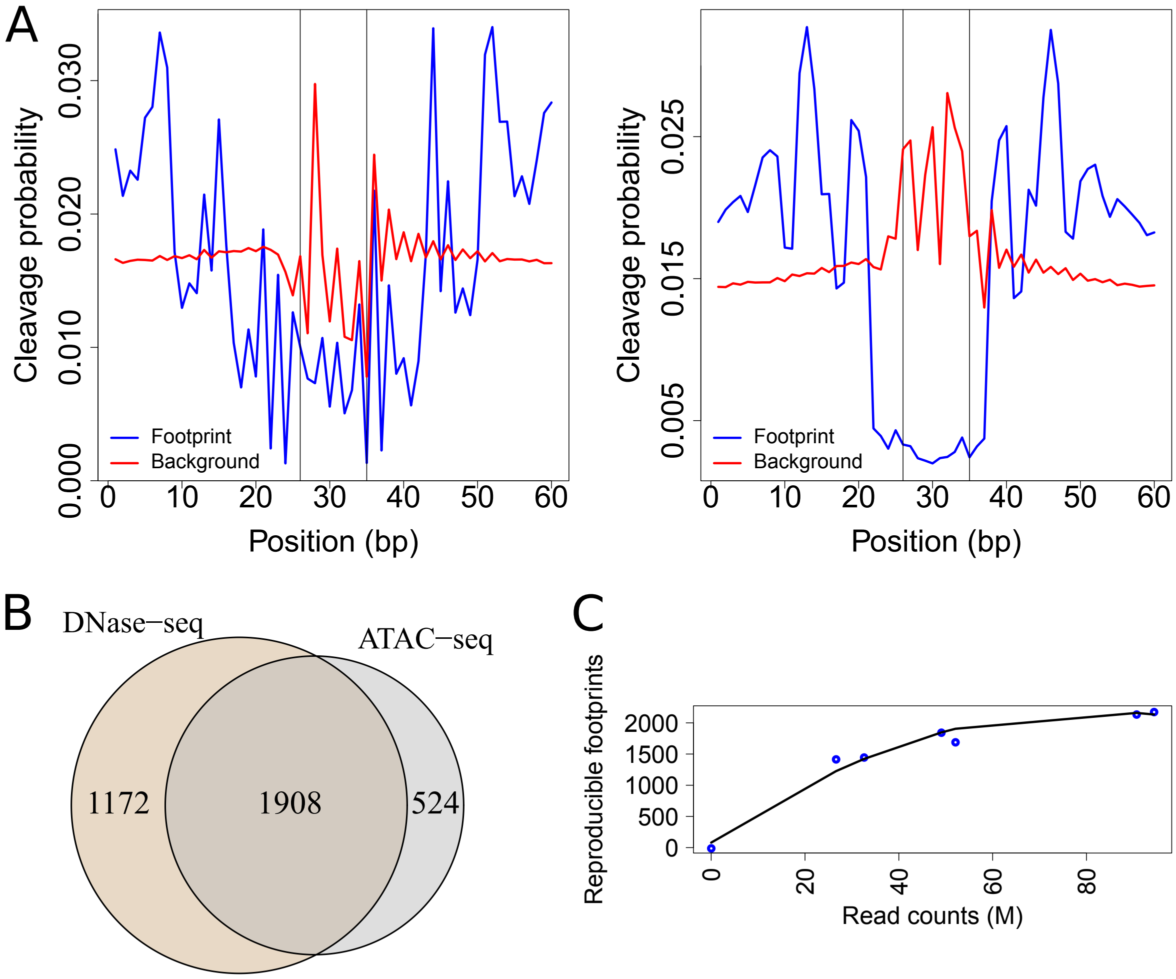

Figure S7: Analysis of NRF1 footprints. (A) NRF1 footprints inferred from K562 ATAC-seq data (left) and DNase-seq data (right). Vertical lines depict the edges of the motif match. (B) Overlap between reproducible NRF1 footprints in the HEK293 DNase-seq and combined ATAC-seq replicates, found using the footprint models learned from the K562 data. (C) Numbers of reproducible NRF1 footprints in HEK293 ATAC-seq datasets at different depths.

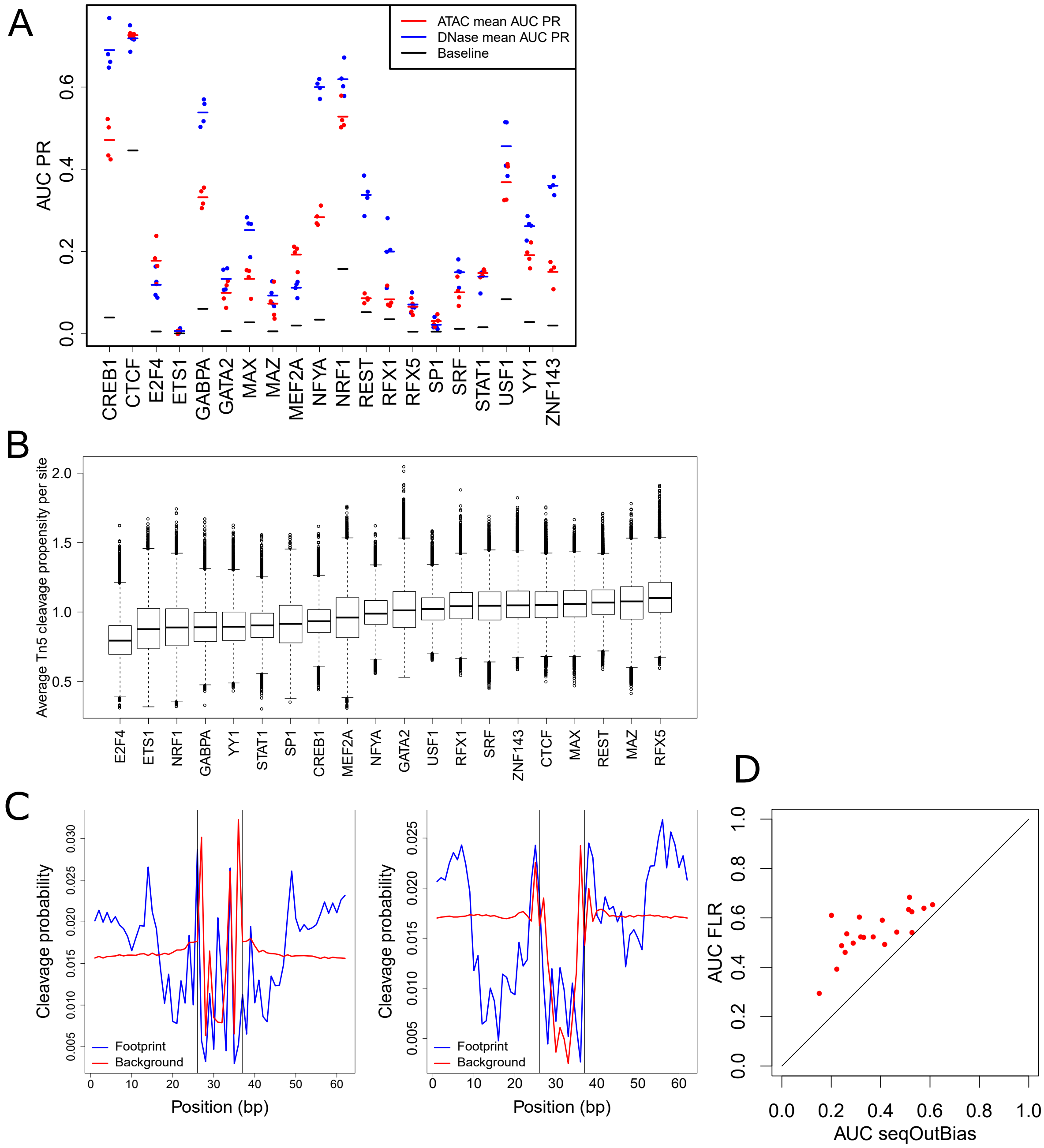

Figure S8: Method and TF-specific footprinting efficiency. (A) Area under the precision-recall curve of footprint models learned for all 20 assayed factors in K562 ATAC-seq and DNase-seq datasets. (B) Average Tn5 cleavage propensities over candidate TFBSs for all 20 assayed factors. (C) MEF2A footprints inferred from K562 ATAC-seq data (left) and DNase-seq data (right). Vertical lines depict the edges of the motif match. (D) Comparison of AUCs (area under the ROC curve) obtained with our method (FLR) vs the seqOutBias method.

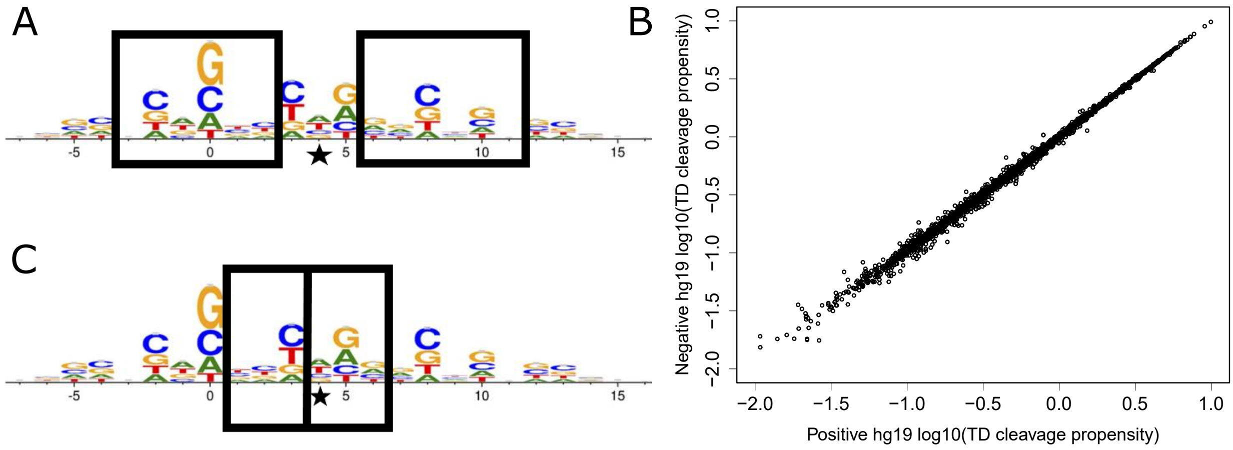

Figure S9: 6-mer bias correction strategy for ATAC-seq datasets. (A) Extended region of sequence bias around Tn5 transposition sites. The 6-mer centered on the cut site and used for bias correction in each read is shown in the left box. The right box shows the 6-mer around the cut site in the opposite strand, 9bps downstream. The center of the 9bp core sequence is marked with a star. (B) Correlation between 6-mer bias values inferred from plus and minus strand reads from libraries generated by Tn5 transposition on deproteinized genomic DNA. (C) As in (A), but showing the 6-mer position upon the conventional +4/-5bp shifting of ATAC-seq reads. Panels (A) and (C) are adapted from reference 40.

Table S1: General statistics of the ATAC-seq datasets generated in the study.

Cell type	Sample description	Total mapped read pairs	Percent mtDNA	Percent uniquely aligned after removing mtDNA	Percent duplication after removing mtDNA	Final read pairs after processing
K562	10 minute lysis	98241437	74.9	60.86	36.1	11824634
K562	5 minute lysis	59725560	73.3	61.68	28.52	8293938
K562	No lysis buffer	64162804	18	76.09	28.83	26203527
HEK293	High depth, bio1-tech1	212332636	21.7	79.15	38.6	74957855
HEK293	High depth, bio1-tech2	215849442	16.7	79.41	42.41	75883012
HEK293	High depth, bio2-tech1	189055455	8.3	80.35	17.42	106390553
HEK293	High depth, bio2-tech2	212178995	3.4	80.84	25.81	112909794
HEK293	Medium depth, bio1-tech1	101177506	22	78.93	22.54	44903594
HEK293	Medium depth, bio1-tech2	115293922	16.9	79.12	27.43	50914321
HEK293	Medium depth, bio2-tech1	85731217	8.4	80.28	8.82	53211877
HEK293	Low depth, bio1-tech1	53199070	21.9	78.99	12.83	26607741
HEK293	Low depth, bio1-tech2	59968056	16.8	79.19	15.84	30798873
HEK293	Low depth, bio2-tech1	40964758	8.4	80.3	4.54	26613414
HEK293	Low depth, bio2-tech2	51835433	3.4	80.81	7.72	34364305

Table S2: Descriptions, accession codes and final read counts for the utilized DNase-seq datasets and libraries generated by Tn5 transposition of deproteinized genomic DNA.

Cell type	Data type	Description	Accession code	Library depth after processing
K562	DNase-seq	Replicate 1 (ENCODE)	ENCFF000SWU	72166285
K562	DNase-seq	Replicate 2 (ENCODE)	ENCFF000SXA	138770111
K562	DNase-seq	Replicate 3 (ENCODE)	ENCFF000SWY	88033023
K562	DNase-seq	Replicate lab	Generated for the study	134851555
HEK293	DNase-seq	Replicate 1 (ENCODE)	ENCFF000SPK	68339552
HEK293	DNase-seq	Replicate 2 (ENCODE)	ENCFF000SQB	164469299
HEK293	DNase-seq	Replicate lab	Generated for the study	126253898
Human (YH1)	Tn5 transposition	Deproteinized genomic DNA	SRX030445	39753928
D. melanogaster	Tn5 transposition	Deproteinized genomic DNA	SRX030438	22705812

Table S3: Scheme for ATAC-seq library comparisons for JAMM-IDR peak calls or FLR-IDR footprint calls.

Comparison name	Biological replicate 1	Biological replicate 2
High depth ATAC-seq 1	High depth, bio1-tech1	High depth, bio2-tech1
High depth ATAC-seq 2	High depth, bio1-tech2	High depth, bio2-tech2
Medium depth ATAC-seq 1	Medium depth, bio1-tech1	Medium depth, bio2-tech1
Medium depth ATAC-seq 2	Medium depth, bio1-tech2	Medium depth, bio2-tech1
Low depth ATAC-seq 1	Low depth, bio1-tech1	Low depth, bio2-tech1
Low depth ATAC-seq 1	Low depth, bio1-tech2	Low depth, bio2-tech2

Table S4: ChIP-seq peaks used in the analysis.

Cell line	Factor	Accession code
HEK293	CTCF	ENCFF002DCV
HEK293	MAZ	ENCFF834ZRT
HEK293	REST	ENCFF201ZGY
HEK293	YY1	ENCFF443TBN
K562	CREB1	ENCFF001UJI, ENCFF001UJJ
K562	CTCF	ENCFF002CEL, ENCFF002CLS, ENCFF002CWL, ENCFF002DBD, ENCFF002DDJ
K562	E2F4	ENCFF002CWM
K562	ETS1	ENCFF002CLX
K562	GABPA	ENCFF002CLZ
K562	GATA2	ENCFF002CMA, ENCFF002CWQ
K562	MAX	ENCFF002CXD
K562	MAZ	ENCFF002CXE
K562	MEF2A	ENCFF002CMD
K562	NFYA	ENCFF002CXI
K562	NRF1	ENCFF002CXK, ENCFF454OVP, ENCFF657YIC, ENCFF664FFU
K562	REST	ENCFF002CMF
K562	RFX1	ENCFF654RTP
K562	RFX5	ENCFF002CXV
K562	SP1	ENCFF002CMN, ENCFF191QSX
K562	SRF	ENCFF002CMP
K562	STAT1	ENCFF002CYB, ENCFF002CYC, ENCFF002CYD, ENCFF002CYE
K562	USF1	ENCFF002CMV
K562	YY1	ENCFF002CMW, ENCFF002CMX, ENCFF002CYQ
K562	ZNF143	ENCFF002CYR

Table S5: PWM IDs used for genome-wide motif searches.

Factor name	PWM ID	Lowest PWM score in top 50K	Closest threshold PWM score	p-value associated with threshold
CREB1	MA0018.2	9.06	7.78555	1*10-6
CTCF	MA0139.1	8.09	7.89799	5*10-5
E2F4	M5180_1.01	1.71	1.78185	2*10-5
ETS1	MA0098.1	8.11	6.9036	1*10-6
GABPA	MA0062.2	8.42	8.43115	4*10-5
GATA2	MA0036.1	7.20	6.24233	1*10-6
MAX	M5613_1.02	5.45	3.68637	1*10-6
MAZ	M00649	9.32	8.22958	1*10-6
MEF2A	M5615_1.02	9.09	4.80812	1*10-6
NFYA	MA0060.1	8.83	8.46208	5*10-5
NRF1	M00652	3.90	1.39346	1*10-6
REST	MA0138.2	5.89	5.80754	3*10-5
RFX1	M00280	8.63	8.53351	5*10-5
RFX5	M5779_1.02	7.03	5.27345	1*10-6
SP1	MA0079.2	9.17	8.38317	1*10-6
SRF	MA0083.1	7.25	6.8771	1*10-6
STAT1	MA0137.2	9.39	9.0732	3*10-5
USF1	M5943_1.02	9.73	9.36591	1*10-6
YY1	M5954_1.02	8.56	7.33829	1*10-6
ZNF143	M5966_1.02	4.96	2.23431	1*10-6
